# Regulation of Neurogenesis by Neurotrophins during Adulthood: Expected and Unexpected Roles

**DOI:** 10.3389/fnins.2016.00026

**Published:** 2016-02-09

**Authors:** Marçal Vilar, Helena Mira

**Affiliations:** ^1^Neurodegeneration Unit, Unidad Funcional de Investigación de Enfermedades Crónicas-Instituto de Salud Carlos IIIMadrid, Spain; ^2^Molecular Neurobiology Unit, Unidad Funcional de Investigación de Enfermedades Crónicas-Instituto de Salud Carlos IIIMadrid, Spain

**Keywords:** adult neurogenesis, neural stem cell, neurotrophin, p75NTR, TrkB, BDNF, NT3

## Abstract

The subventricular zone (SVZ) of the anterolateral ventricle and the subgranular zone (SGZ) of the hippocampal dentate gyrus are the two main regions of the adult mammalian brain in which neurogenesis is maintained throughout life. Because alterations in adult neurogenesis appear to be a common hallmark of different neurodegenerative diseases, understanding the molecular mechanisms controlling adult neurogenesis is a focus of active research. Neurotrophic factors are a family of molecules that play critical roles in the survival and differentiation of neurons during development and in the control of neural plasticity in the adult. Several neurotrophins and neurotrophin receptors have been implicated in the regulation of adult neurogenesis at different levels. Here, we review the current understanding of neurotrophin modulation of adult neurogenesis in both the SVZ and SGZ. We compile data supporting a variety of roles for neurotrophins/neurotrophin receptors in different scenarios, including both expected and unexpected functions.

## Introduction

Neurotrophins (NTs) are implicated in the maintenance and survival of the peripheral and central nervous systems and mediate several forms of synaptic plasticity (Chao, 2003; Ceni et al., 2014; Hempstead, 2014; Lu et al., 2014). Nerve growth factor (NGF) was the first discovered member of the family (Cohen et al., 1954), which also includes brain-derived neurotrophic factor (BDNF), neurotrophin-3 (NT3), and neurotrophin 4/5 (NT4/5) (reviewed recently in Bothwell, 2014). Neurotrophins were first identified as survival factors for developing neurons, but are pleiotropic molecules that can exert a variety of functions, including the regulation of neuronal differentiation, axonal and dendritic growth, and synaptic plasticity (Bothwell, 2014).

NTs interact with two distinct receptors, a cognate member of the Trk receptor tyrosine kinase family and the common p75 neurotrophin receptor (p75NTR), which belongs to the tumor necrosis factor receptor (TNFR) superfamily of death receptors (Friedman and Greene, 1999; Huang and Reichardt, 2003). Tropomyosin receptor kinase (Trk) receptors belong to the family of receptor tyrosine kinases (reviewed in Deinhardt and Chao, 2014) and contain an extracellular domain at which the NT binds, a single transmembrane domain, and an intracellular domain (ICD) with tyrosine kinase activity. Three different Trks have been identified in mammals: TrkA, TrkB, and TrkC. NGF is the preferred ligand for TrkA, BDNF, and NT4/5 are preferred for TrkB, and NT3 for TrkC. These specificities are not absolute, and NT3 is also a ligand for TrkA and TrkB. In addition to full length (FL) receptors, splice variants containing either deletions in the extracellular domain or intracellular truncations including the kinase domain have been described. These receptor molecules may influence ligand specificity, restrict its availability by internalization, or act as dominant-negative modulators. Splice variants of TrkB, designated T1 and T2, are expressed at high levels in the mature brain (Klein et al., 1990).

While Trk receptors bind only to mature neurotrophins, p75NTR also interacts with pro-neurotrophins, increasing the complexity of its signaling. Upon pro-NGF binding, cell death is induced by a complex consisting of p75NTR and sortilin (Hempstead, 2014). Similarly, pro-BDNF induces axon pruning of hippocampal neurons in culture (Hempstead, 2015). Although, Trk signaling is involved in survival and differentiation (Reichardt, 2006; Deinhardt and Chao, 2014), p75NTR participates in several signaling pathways (Kraemer et al., 2014) governed by the cell context and the formation of complexes with different co-receptors and ligands, such as sortilin/pro-NGF in cell death (Nykjaer et al., 2004) and Nogo/Lingo-1/NgR in axonal growth (Wang et al., 2002; Mi et al., 2004). p75NTR also undergoes shedding and receptor intramembrane proteolysis (RIP), resulting in the release of its ICD, which itself possesses signaling capabilities related to migration, proliferation, and transcriptional modulation (Jung et al., 2003; Kanning et al., 2003; Skeldal et al., 2012). Two different isoforms of p75NTR have been described, a long isoform and a short isoform (Naumann et al., 2002). These isoforms differ based on the presence or absence of the NT binding domain, respectively.

In recent years, NTs and their receptors have emerged as important regulators of adult neurogenesis. The production of new neurons persists throughout life in two regions or niches of the mammalian brain in which neurotrophins are also present: the subependymal or subventricular zone (SVZ) adjacent to the lateral ventricles, and the subgranular zone (SGZ) of the dentate gyrus in the hippocampal formation. Neurogenesis persists in these specialized areas owing to the existence of a population of neural stem cells (NSCs) that retain the capacity to proliferate and generate new neurons via a series of intermediate progenitor cells. NSCs can also give rise to glial cells. In both the SVZ and SGZ, these NSCs are largely regulated by local signals emanating from other niche cell types such as astrocytes or endothelial cells. In addition, SVZ-NSCs contact the ventricular lumen and are regulated by signals within the cerebral spinal fluid (CSF), and by signals released by ependymal cells. Neuroblasts and immature neurons born in the SVZ are exposed to signals from the rostral migratory stream (RMS) during their journey to the olfactory bulb (OB), where they terminally integrate.

Here, we review the current understanding of the role of neurotrophins and their receptors in the regulation of adult neurogenesis, as well as the underlying mechanisms. We discuss the main findings pertaining to the two main neurogenic niches of the adult brain, the SVZ and the SGZ. We also highlight unexpected findings that have expanded the traditional perspective of neurotrophin function.

## Expression pattern of neurotrophins and their receptors in the neurogenic niches of the adult SVZ and SGZ

**Figure 1:**
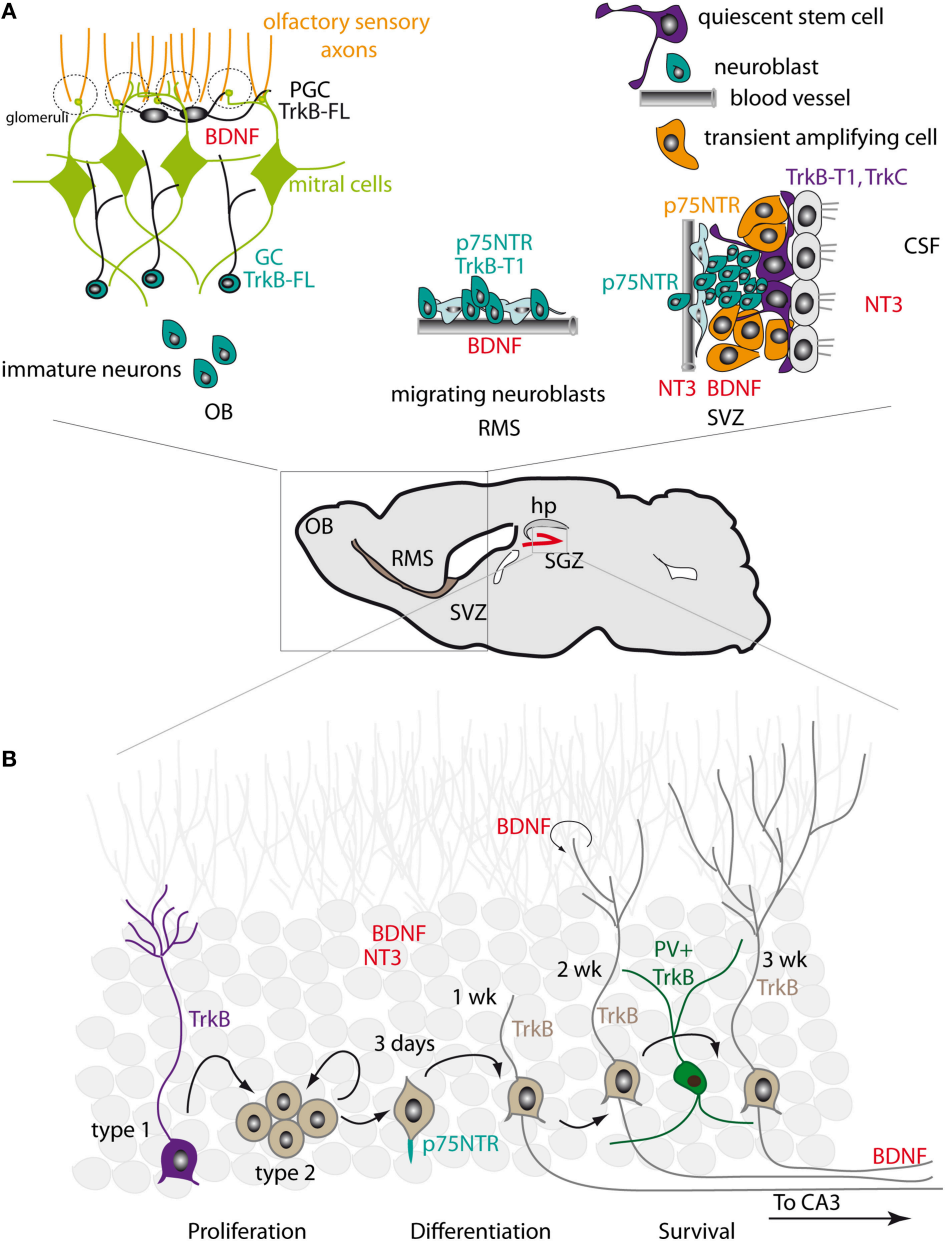
**Expression patterns of neurotrophins and their receptors in the adult SVZ-RMS-OB and SGZ. (A)** *Right*. In the SVZ, NT3 is released into the CSF by the choroid plexus and is produced by the endothelial cells of blood vessels. BDNF is also present in the SVZ. Truncated TrkB-T1 is expressed in ependymal cells and in NSCs. NSCs also express TrkC. p75NTR is found in transient amplifying intermediate progenitors and in neuroblasts. *Middle*. In the RMS, BDNF is synthesized by the endothelial cells of blood vessels that outline the migratory stream. Neuroblasts express truncated TrkB-T1 and p75NTR. *Left*. In the OB, BDNF is concentrated in the mitral and glomerular layers. Immature neurons entering the OB acquire the expression of TrkB-FL, which is detected in granule cells (GC) and in periglomerular cells (PGC). **(B)** In the dentate gyrus, high levels of NT3 and BDNF are produced by mature neurons. BDNF is also produced by newly generated neurons. Mossy fiber axons of dentate granule neurons display strong BDNF immunoreactivity. TrkB is expressed in NSCs with radial morphology (Type 1 cells). Proliferating progenitors (Type 2 cells) have low TrkB levels. Young neurons and more mature granule neurons re-acquire high levels of TrkB expression. Parvalbumin (PV)-positive interneurons also express TrkB. p75NTR is expressed in newborn cells initiating axon growth, before entry of the axon fibers to the CA3 area, and is enriched at the initiation site of the axon fiber.

The expression of NTs by local cells in the rodent SVZ niche is generally very low, although some ligands such as BDNF can be detected by immunohistochemistry (Figure 1A; Galvao et al., 2008). In addition, several NTs are expressed in the choroid plexus (CP), which secretes CSF components that become readily accessible to SVZ-NSCs, the so-called type B cells. The most abundantly expressed neurotrophin in the CP is NT4 (Galvao et al., 2008). NT3 is also released by CP capillaries and by endothelial cells (Delgado et al., 2014). In primates, NGF and BDNF are expressed by SVZ astrocytes while NT3 is found in ependymal cells (Tonchev, 2011). BDNF is mainly detected in the rostral migratory stream (RMS) and the OB (Maisonpierre et al., 1990; Bath et al., 2008; Galvao et al., 2008; Snapyan et al., 2009; Bagley and Belluscio, 2010). In the RMS, BDNF is synthesized by the endothelial cells of blood vessels that outline the migratory stream for new neurons (Snapyan et al., 2009). In the OB, the BDNF ligand is largely concentrated in the mitral and glomerular layers (Bergami et al., 2013). The cellular source of BDNF in the OB is yet to be clarified. Recently, Bergami et al. suggested that both local glutamatergic neurons (mitral and tufted cells) and long projecting neurons from the anterior olfactory nucleus (AON) and piriform cortex (PC) are the main sources of BDNF in the OB (Bergami et al., 2013).

The expression pattern of neurotrophin receptors in the adult mouse SVZ, as revealed by RT-PCR (Galvao et al., 2008), indicates that TrkB is the most abundant receptor, although TrkC is also present (Figure 1A). Despite some controversy, the prevailing view is that in both the mouse and rat SVZ truncated TrkB-T1 is expressed in ependymal cells and in type B cells (Chiaramello et al., 2007; Bath et al., 2008). This includes the BrdU-retaining cells that correspond to the slowly dividing NSCs. Type B NSCs also express TrkC, but not TrkA (Delgado et al., 2014). Maturing neuroblasts in the RMS appear to express the truncated form of TrkB, but express TrkB-FL upon entering the OB (Kirschenbaum and Goldman, 1995; Gascon et al., 2005; Bath et al., 2008; Snapyan et al., 2009; Bagley and Belluscio, 2010; Bergami et al., 2013). By contrast, p75NTR is expressed in intermediate progenitors (type C cells) of the SVZ and in neuroblasts (type A cells) of the SVZ/RMS (Galvao et al., 2008). Intriguingly, in rats a larger proportion of SVZ cells express p75NTR, although this receptor does not significantly co-localize with type B markers such as GFAP and appears to be largely confined to EGFR^+^ type C progenitor cells, with some low level expression in PSA-NCAM^+^ or DCX^+^ neuroblasts (Giuliani et al., 2004; Young et al., 2007). This suggests that p75NTR may play a greater role in the regulation of SVZ progenitor proliferation in rats than in mice, and that neurotrophin signaling in the two species may differ slightly. In primates, TrkA is present in SVZ astrocytes and immature neurons, TrkB is detected in astrocytes and neural progenitors, and TrkC is found in ependymal cells (Tonchev, 2011).

Neurotrophic factors and their receptors are abundantly expressed in the hippocampus, with an expression pattern that differs markedly to that of the SVZ (Figure 1B). For instance, NT3 is produced at very high levels in the dentate gyrus and is mainly expressed in neurons (Maisonpierre et al., 1990; Lauterborn et al., 1994; Shimazu et al., 2006). BDNF is also strongly expressed (Li et al., 2008). Both BDNF mRNA and protein expression are particularly high, with mossy fiber axons of dentate granule neurons displaying strong BDNF immunoreactivity due to anterograde transport (Conner et al., 1997). BDNF is also likely expressed in non-neuronal cells (Murer et al., 2001). In the adult macaque brain, the highest levels of BDNF are found in the hippocampus, indicating that BDNF also plays an important role in SGZ neurogenesis in non-murine species (Mori et al., 2004). TrkB appears to be broadly expressed: its expression is high in NSCs with radial morphology and low in proliferating progenitors, while young DCX^+^ neurons and more mature granule neurons re-acquire high levels of TrkB expression (Donovan et al., 2008). By contrast, p75NTR expression is observed in a very narrow time window during the course of SGZ neurogenesis. Retroviral tracing experiments indicate that p75NTR expression is mainly confined to newborn cells between 3 and 7 days after retroviral injection (Zuccaro et al., 2014); these correspond to cells initiating the growth of the axon and dendritic processes, before axonal fibers reach the CA3 area (Zhao et al., 2006). At this stage, p75NTR is asymmetrically enriched at the initiation site of axon fibers and in proximal axon segments. In more mature neurons p75NTR expression is decreased and the asymmetric distribution of the receptor is lost (Zuccaro et al., 2014). Interestingly, p75NTR (but not TrkA) appears to be also localized in the primary cilia of dentate gyrus granule neurons, a specialized structure that acts as a sensory organelle and is involved in signal transduction (Chakravarthy et al., 2010).

## Functional significance of neurotrophins and their receptors in the SVZ

The few studies that have analyzed the effects of BDNF in the postnatal and adult SVZ have yielded mixed results. Galvao and coworkers reported that BDNF infusion reduces SVZ proliferation in both mice and rats. However, species-specific differences do exist; BDNF infusion and adenoviral vector-mediated overexpression of BDNF have no effect on OB neurogenesis in mice, or attenuate it in the long term (Galvao et al., 2008; Reumers et al., 2008), but have been shown in some studies to increase the number of new OB neurons in rats (Zigova et al., 1998; Benraiss et al., 2001; Henry et al., 2007).

Abundant data implicate BDNF in neuroblast migration along the RMS, although it remains to be established which receptor (TrkB-FL, TrkB-T1, or p75NTR) mediates this activity. In acute slice preparations, BDNF expressed by endothelial cells promotes neuronal migration along the RMS via p75NTR expressed on neuroblasts (Snapyan et al., 2009). BDNF/TrkB signaling may also contribute to the modulation of neuroblast migration (Bagley and Belluscio, 2010). However, grafting studies in which SVZ cells from TrkB knockout (TrkB-KO) and wild type (WT) mice were transplanted into the SVZ of adult WT mice suggest that TrkB is not essential at a cell-autonomous level for the migration of newly generated OB neurons (Galvao et al., 2008). In line with these observations, Bergami and colleagues observed no changes in the RMS migration of adult-born neurons from TrkB^lox∕lox^ mice, in which the TrkB-FL is deleted in progenitors by Cre expression (Bergami et al., 2013).

BDNF and TrkB participate in the long term survival and maturation of specific interneuron subpopulations of the OB. Berghuis et al. reported that loss of BDNF decreases the number and complexity of parvalbumin^+^ (PV^+^) cells in the external plexiform layer (Berghuis et al., 2006). Other authors have shown that periglomerular tyrosine hydroxylase^+^ (TH^+^) dopaminergic neurons are more sensitive to TrkB loss (Galvao et al., 2008; Bergami et al., 2013). TrkB deletion compromises the survival of TH^+^ neurons, an effect that is accompanied by an increase in the number of calretinin^+^ (CR^+^) neurons, suggesting that TrkB regulates the balance between different classes of adult-born neurons (Bergami et al., 2013). As mentioned above, both local and long projecting neurons are sources of BDNF in the OB (Bergami et al., 2013). This may explain the existence of a BDNF expression gradient, which results in differences in BDNF availability, and the heterogeneous distribution of apoptotic TrkB-KO neurons in the OB (Bergami et al., 2013). Experiments with the mutant BDNF variant Val66Met suggest that activity-dependent BDNF release is required to ensure the survival of newly born OB neurons (Bath et al., 2008).

Using conditional knockout mice, Bergami and coworkers performed an in-depth analysis of the late role of TrkB-FL in OB neurogenesis. They elegantly showed that while newborn TrkB-FL-deficient neurons of the granule cell layer (GCL) have normal dendritic trees, the spine density of these neurons is compromised. By contrast, in newborn TH^+^, TrkB-FL-deficient neurons of the periglomerular cell layer both dendritic arborization and spine growth are impaired, further highlighting the TrkB-dependency of this neuronal subpopulation. Notably, phospholipase C-γ signaling appears to mediate the effects of TrkB on spine growth, while Shc/PI3K signaling is involved in dendritic branching (Bergami et al., 2013).

The role of p75NTR in the SVZ remains to be established. p75NTR^+^ cells are present in the SVZ and in the RMS in young and adult animals (Giuliani et al., 2004; Gascon et al., 2007; Young et al., 2007; Galvao et al., 2008). The Bartlett group identified a small population of precursor cells that express high levels of p75NTR and respond to BDNF (Young et al., 2007), in agreement with previous findings (Bibel et al., 2004). In p75NTR^ExIII^-knockout animals, which lack the full-length receptor but express the short isoform, these authors observed a reduction in the number of PSA-NCAM^+^ SVZ migrating neuroblasts and a decrease in OB size *in vivo*, suggesting a role of p75NTR in the migration and possibly survival of mature neurons in the OB (Young et al., 2007). However, these findings were subsequently challenged by those of another group (Bath et al., 2008), who failed to detect such differences. A recent study reported that p75NTR is expressed in migrating neuroblasts and responds to vasculature-secreted BDNF (Snapyan et al., 2009). Taken together, the available data suggest that p75NTR plays a dual role in OB neurogenesis, regulating proliferation in the SVZ by controlling the production of neuronal precursors (Young et al., 2007; Bath et al., 2008), and regulating migration of RMS neuroblasts en route to the OB (Snapyan et al., 2009).

In addition to neurotrophin signaling via p75NTR, amyloid peptide Aβ appears to regulate the proliferation of neural progenitors and adult neurogenesis via p75NTR (Sotthibundhu et al., 2009). Exogenous and endogenous stimulation of SVZ precursor cells by Aβ promote neurogenesis (Sotthibundhu et al., 2009). This effect is dependent on p75NTR expression by precursor cells, and can be blocked by preventing the proteolytic processing of p75NTR (Sotthibundhu et al., 2009). Overstimulation of p75NTR^+^ neural progenitor cell division by Aβ in early life may result in premature depletion of the stem cell pool and a rapid decline in basal neurogenesis later in life (Sotthibundhu et al., 2009), as shown for instance in models of aging in which Aβ levels are increased (Diaz-Moreno et al., 2013).

Recent studies have shown that the role of neurotrophins in adult neurogenesis is even more complex than previously thought. Delgado and coworkers revealed an unprecedented role of NT3 in regulating the quiescent state of adult SVZ-NSCs (Delgado et al., 2014). They found that NT3 released by brain endothelial cells and choroid plexus capillaries is bound by NSCs, promoting their quiescence. Furthermore, by infusing NT3 into the lateral ventricle they obtained evidence suggesting that NSCs take up the neurotrophin via receptors located in their primary cilium, which acts as a sensor for ligands circulating in the CSF. The proliferative rate of NSCs was increased in young (2-month-old) Ntf3 heterozygous mice, in which NT3 expression is reduced. These mice also showed increased numbers of BrdU-retaining stem cells. A similar phenotype was observed in conditional Ntf3 knockout mice in which NT3 expression is specifically abolished in blood vessels. Interestingly, the cell fate of NSC progeny appeared not to depend on NT3 levels; the proportion of newly generated oligodendrocytes in the corpus callosum and that of newly generated neurons in the OB were similarly increased in Ntf3 heterozygous mice in response to the increase in NSC activity. In line with this observation, the authors detected increases in all specific OB interneuron subpopulations analyzed, further supporting the view that neuronal subtype identity is independent of NT3. In older (8-month-old) animals, NSCs prematurely differentiated into astrocytes, leading to NSC loss. These findings suggest that a decrease in the availability of NT3 results first in hyperproliferation, followed by premature exhaustion of the NSC pool. From a mechanistic standpoint, NT3 activates the nitric oxide synthase isoform eNOS in NSCs, resulting in production of the second messenger NO and cytostasis.

## Functional significance of neurotrophins and their receptors in the SGZ

In the dentate gyrus NT3 facilitates learning and memory, possibly by stimulating neuronal differentiation and/or the survival of newly born cells (Shimazu et al., 2006). Conditional Ntf3-knockout mice in which the gene encoding NT3 is deleted in the brain throughout development show normal proliferation in the SGZ, a reduction in the number of newly generated NeuN^+^ granule neurons, and an increase in the proportion of cells that do not express differentiation markers, pointing to a role of NT3, and perhaps also its preferred receptor TrkC, in maturation (Shimazu et al., 2006).

The p75NTR receptor also regulates hippocampal neurogenesis (Catts et al., 2008). In p75NTR^ExIII^-knockout animals, which lack the full-length receptor but express the short p75NTR isoform, a reduction in the number of neuroblasts and newborn neurons in the dentate gyrus is paralleled by an increase in the death of newly born cells and impaired performance of hippocampus-dependent behavioral tasks (Catts et al., 2008). However, p75NTR^ExIV^-knockout mice, in which both the long and short isoforms are deleted, show an increase in the number and complexity of DCX^+^ newborn neurons and a decrease in cell death (Poser et al., 2015). These contradictory findings may be explained by the differing levels of expression of the short isoform between the two mouse models, although a detailed study of the expression of the short isoform in the mouse hippocampus has yet to be performed. The artifactual overexpression of the p75 ICD in p75NTR^ExIV^ knockout mice (Paul et al., 2004) may also explain the different hippocampal phenotypes found in the two p75NTR-knockout strains. The analysis of adult neurogenesis in newly developed conditional mouse models, in which both the short and long p75NTR isoforms are cleanly deleted (Boskovic et al., 2014; Zuccaro et al., 2014), may shed some light on these discrepant findings. A study of p75^lox∕lox^ animals by Zuccaro and coworkers focused on axon growth, and demonstrated that polarized expression of p75NTR specifies the future axon in newly generated neurons of the adult SGZ (Zuccaro et al., 2014). These authors also found that the injection of lentiviruses transducing shRNA against p75NTR increases the proportion of new neurons lacking an axon, and that axogenesis in p75^lox∕lox^ animals is impaired by the conditional deletion of the receptor in progenitors.

The overall importance of BDNF/TrkB in adult hippocampal neurogenesis is clear. For example, neurogenesis is attenuated by BDNF knockdown in the dentate gyrus using lentiviral-mediated RNAi (Taliaz et al., 2010), but increased in response to exogenous BDNF injection (Scharfman et al., 2005). Nonetheless, there is less of a consensus as regards the participation of BDNF/TrkB in certain aspects of neurogenesis, such as the proliferation of progenitor cells and the survival of new neurons. TrkB is required for normal proliferation and neurogenesis in the SGZ, although conflicting results have been reported. Conditional deletion of TrkB in hippocampal NSCs reduces SGZ proliferation in postnatal day 15 (P15) animals and in adults, but has no effect on overall cell survival (Li et al., 2008). Animals with impaired TrkB activation (TrkB-T1-overexpressing mice) display an increase in proliferation and a reduction in survival (Lee et al., 2002; Sairanen et al., 2005). *In vitro*, BDNF promotes the proliferation of hippocampal neural progenitor cultures in a TrkB-dependent manner (Li et al., 2008).

In BDNF germline heterozygous mice, both increases (Sairanen et al., 2005) and decreases (Lee et al., 2002) in proliferation have been reported, as measured 1 day after BrdU injection. Studies using conditional knockout mice in which mature hippocampal neurons lack the BDNF gene have also been inconclusive, with some authors describing increased proliferation of SGZ progenitor cells (Chan et al., 2008) and others reporting no alteration (Choi et al., 2009). These conflicting results have not yet been explained, although it is possible that developmental and/or behavioral differences between the strains used in the aforementioned studies may contribute to the divergent findings. The functional role of BDNF in the survival of new neurons in the adult dentate gyrus is a matter of some debate. Impairment of basal levels of survival in BDNF heterozygous mice has been reported in some studies (Sairanen et al., 2005) but not in others (Rossi et al., 2006). Survival of newborn cells is also slightly attenuated in mice in which BDNF is deleted in granule neurons (Choi et al., 2009; Gao et al., 2009).

A greater consensus has been reached however regarding the role of BDNF/TrkB signaling in dendrite morphogenesis in newborn SGZ neurons (Bergami et al., 2008; Wang et al., 2015). Dendrite and spine growth is markedly altered in adult-born granule neurons of TrkB^lox∕lox^ mice, in which TrkB-FL is deleted in progenitors via Cre expression (Bergami et al., 2008). Moreover, TrkB-deficient neurons show impaired synaptic plasticity, and a proportion of these newly generated neurons die during the transition from immature to more mature stages. In line with these findings, a dramatic reduction in dendritic spine density has been described in the dentate gyrus of BDNF heterozygous mice (Zhu et al., 2009), and dendritic development, synaptic formation, and synaptic maturation are all impaired in postnatal-born granule neurons in BDNF conditional knockout mice (Gao et al., 2009). BDNF has also been shown to regulate late phases of neuronal differentiation, and dendritic development of adult-generated granule neurons is compromised in BDNF conditional mutants (Chan et al., 2008).

A recent study showed that dendrite growth is decreased in response to BDNF deletion in adult-born hippocampal neurons using retroviral vectors, and increased by BDNF overexpression (Wang et al., 2015). This effect appears to be largely autocrine, as BDNF deletion in newborn neurons only gives rise to dendritic abnormalities similar to those observed in conditional knockout mice in which BDNF is deleted throughout the entire forebrain. This is consistent with the full restoration of normal dendritic development in adult-born cells of BDNF conditional knockouts in which BDNF is selectively re-expressed (Wang et al., 2015). The autocrine production of the neurotrophin may depend on neuronal activity due to excitatory synaptic inputs onto the developing dendrites of the newborn neurons, or due to the earlier excitatory action of GABA released by interneurons (Wang et al., 2015). Interestingly, BDNF expression is highly complex and the protein can be translated from different mRNA species harboring either a short or a long 3′ untranslated region (3′ UTR), the latter of which targets BDNF mRNA to dendrites for local translation. BDNF translated from this long 3′ UTR mRNA in dendrites indirectly promotes the maturation of adult-born neurons, via GABAergic interneurons in the dentate gyrus (Waterhouse et al., 2012). Bdnf^klox∕klox^ mice, in which the long 3′ UTR is truncated, show increased progenitor proliferation and impaired differentiation and maturation of newborn hippocampal neurons. Similar results have been obtained in mice with selective deletion of TrkB in parvalbumin (PV)-expressing GABAergic interneurons. These results indicate that locally synthesized BDNF in the dendrites of granule neurons promotes differentiation and maturation of progenitor cells in the SGZ at least in part by enhancing GABA release from PV^+^ GABAergic interneurons (Waterhouse et al., 2012).

A fascinating aspect of the regulation of adult hippocampal neurogenesis by NTs is the connection between BDNF and the modulation of hippocampal neurogenesis by external stimuli, a topic that has been extensively studied in recent years (reviewed recently in Vivar et al., 2013 and Aimone et al., 2014). Adult neurogenesis in the dentate gyrus is enhanced by voluntary exercise, exposure to an enriched environment, and chronic antidepressant administration. Interestingly, many studies have shown that physical exercise increases hippocampal expression of BDNF (and NGF, but apparently not NT3; Cotman and Berchtold, 2002; Berchtold et al., 2005; Vaynman and Gomez-Pinilla, 2005; Cotman et al., 2007; Vivar et al., 2013). This increase correlates with the beneficial effects of exercise. For instance, long-term voluntary running increases BDNF levels while improving spatial memory and hippocampal neurogenesis (Marlatt et al., 2012). Eight months of forced exercise prevents the age-related impairments in both plasticity and BDNF expression in the dentate gyrus (O'Callaghan et al., 2009). Five weeks of treadmill running increases BDNF and TrkB expression, enhances NSC proliferation, and promotes the maturation and survival of immature neurons (Wu et al., 2008). TrkB ablation in adult hippocampal NSCs also blocks the effect of voluntary exercise on proliferation and neurogenesis (Li et al., 2008). Specific deletion of BDNF in mature hippocampal neurons has only a modest impact on the exercise-mediated increase in SGZ proliferation, suggesting that additional sources of BDNF are involved in this process (Choi et al., 2009). Interestingly, BDNF deletion in adult-born granule cells abolishes the increase in dendritic growth induced by running (Wang et al., 2015). This suggests that the effects of exercise on dendritic growth depend on autocrine BDNF signaling occurring in the newly generated neurons.

Environmental enrichment (EE) also increases hippocampal BDNF levels (but not NGF expression) in long term but not short term EE paradigms (Ickes et al., 2000; Kuzumaki et al., 2011). Up-regulation of BDNF in EE is caused by histone modifications of the BDNF promoter (Kuzumaki et al., 2011). As regards the role of this neurotrophin in EE, it has been reported that hippocampal neurogenesis is not increased in BDNF heterozygous mice placed in an enriched environment for 8 weeks. In the same EE setup, wild-type and NT4-knockout mice show a two-fold increase in hippocampal neurogenesis, pointing to BDNF as a central player in the EE-mediated induction of neurogenesis (Rossi et al., 2006). However, more recent studies have challenged this view. The positive effect of EE on proliferation is unaffected in a different model using mice with reduced hippocampal BDNF levels, such as those with conditional ablation of BDNF in mature hippocampal neurons (Choi et al., 2009). Moreover, conditional deletion of BDNF in mature neurons impairs dendritic development of DCX^+^ immature neurons in mice in standard housing (Chan et al., 2008; Choi et al., 2009), a defect rescued by EE (Choi et al., 2009). Altered dendritic spine density in the dentate gyrus of BDNF heterozygous mice is also partly rescued by EE (Zhu et al., 2009), suggesting that EE can modulate dendritic development even in mice with low BDNF levels. Recent findings suggest that BDNF levels are increased in EE only when running wheels are accessible to the animals, indicating that physical exercise may be the critical factor required to trigger BDNF overexpression in EE (Kobilo et al., 2011).

Antidepressants also increase BDNF/TrkB expression in the hippocampus (Nibuya et al., 1995; Russo-Neustadt et al., 2000; Sairanen et al., 2005), and their behavioral effects are mimicked by BDNF infusion (Shirayama et al., 2002). TrkB ablation in adult hippocampal NSCs blocks the increase in proliferation and neurogenesis that occurs in response to antidepressants (Li et al., 2008). Moreover, chronic antidepressant administration impairs the survival of newly generated SGZ neurons in BDNF heterozygous mice and in transgenic mice expressing the dominant negative TrkB-T1 isoform (Sairanen et al., 2005).

## Concluding remarks

In recent years a large number of studies have investigated the role of NTs in adult neurogenesis, identifying both expected and unexpected functions for this protein family of ligands and their receptors. A recurrent theme has been the prominent role of BDNF/TrkB in dendrite and spine morphogenesis, both in the OB and the dentate gyrus. Furthermore, p75NTR has been linked to progenitor proliferation and migration in the SVZ-RMS and to axon initiation in the SGZ. NT3 appears to play a very early role in the SVZ as a modulator of NSC quiescence and a late role in the dentate gyrus, where it likely regulates maturation/survival of newly generated neurons. Despite these advances, several key questions remain unanswered. For instance, the function of pro-neurotrophins and their regulation by external stimuli has been scarcely addressed. Cross-talk between NTs and other niche factors also remains largely unexplored. More studies are clearly needed to fully understand the signaling pathways operating downstream of NTs and their receptors, and to determine whether or not these pathways are altered in pathological processes. We anticipate that future research will add to and further refine current knowledge, and possibly uncover additional functions for neurotrophins in adult neurogenesis, both in health and disease.

## Author contributions

All authors listed, have made substantial, direct, and intellectual contribution to the work, and approved it for publication.

### Conflict of interest statement

The authors declare that the research was conducted in the absence of any commercial or financial relationships that could be construed as a potential conflict of interest.
